# Endogenous cholinergic tone modulates spontaneous network level neuronal activity in primary cortical cultures grown on multi-electrode arrays

**DOI:** 10.1186/1471-2202-14-38

**Published:** 2013-03-26

**Authors:** Mark W Hammond, Dimitris Xydas, Julia H Downes, Giovanna Bucci, Victor Becerra, Kevin Warwick, Andrew Constanti, Slawomir J Nasuto, Benjamin J Whalley

**Affiliations:** 1 School of Chemistry, Food and Nutritional Sciences and Pharmacy, University of Reading, Whiteknights, Reading, Berkshire, RG6 6AP, UK; 2 School of Systems Engineering, University of Reading, Whiteknights, Reading, Berkshire, RG6 6AP, UK; 3 UCL School of Pharmacy, 29-39 Brunswick Square, London, WC1N 1AX, UK

**Keywords:** Acetylcholine, Endogenous cholinergic tone, Cortical culture, Multi-electrode array

## Abstract

**Background:**

Cortical cultures grown long-term on multi-electrode arrays (MEAs) are frequently and extensively used as models of cortical networks in studies of neuronal firing activity, neuropharmacology, toxicology and mechanisms underlying synaptic plasticity. However, in contrast to the predominantly asynchronous neuronal firing activity exhibited by intact cortex, electrophysiological activity of mature cortical cultures is dominated by spontaneous epileptiform-like global burst events which hinders their effective use in network-level studies, particularly for neurally-controlled animat (‘artificial animal’) applications. Thus, the identification of culture features that can be exploited to produce neuronal activity more representative of that seen *in vivo* could increase the utility and relevance of studies that employ these preparations. Acetylcholine has a recognised neuromodulatory role affecting excitability, rhythmicity, plasticity and information flow *in vivo* although its endogenous production by cortical cultures and subsequent functional influence upon neuronal excitability remains unknown.

**Results:**

Consequently, using MEA electrophysiological recording supported by immunohistochemical and RT-qPCR methods, we demonstrate for the first time, the presence of intrinsic cholinergic neurons and significant, endogenous cholinergic tone in cortical cultures with a characterisation of the muscarinic and nicotinic components that underlie modulation of spontaneous neuronal activity. We found that tonic muscarinic ACh receptor (mAChR) activation affects global excitability and burst event regularity in a culture age-dependent manner whilst, in contrast, tonic nicotinic ACh receptor (nAChR) activation can modulate burst duration and the proportion of spikes occurring within bursts in a spatio-temporal fashion.

**Conclusions:**

We suggest that the presence of significant endogenous cholinergic tone in cortical cultures and the comparability of its modulatory effects to those seen in intact brain tissues support emerging, exploitable commonalities between *in vivo* and *in vitro* preparations. We conclude that experimental manipulation of endogenous cholinergic tone could offer a novel opportunity to improve the use of cortical cultures for studies of network-level mechanisms in a manner that remains largely consistent with its functional role.

## Background

Despite lacking a genetically defined, layered or ‘cell nucleus’ topology, primary neuronal cultures share many features with the tissues from which they are obtained, including cell phenotypes, receptor and ion channel complements, intrinsic electrical membrane properties, synaptic development and plasticity [1-4]. As such, cultures have been widely used as models of *in vivo* networks in numerous study types, including neuronal firing dynamics during development [5], neuropharmacology [6], neurotoxicity [7,8], disease states such as Alzheimer’s [9] and, most recently, the study of information flow and synaptic plasticity within networks [10-15]. However, the dominant mode of spontaneous neuronal activity exhibited by such cultures, including cortical cultures, is recurrent, high frequency, synchronised activity (termed ‘global bursts’ [16,17]) which, outside of hypersynchrony diseases such as epilepsy [18], early periods of synaptic development [19] and slow wave sleep [20], is rarely seen *in vivo*.

In addition to being unrepresentative, the presence of spontaneous global bursting interferes with experimental aims, confounds findings and so hinders the translation of results obtained from cultures to *in vivo* conditions. For example, conflicting results have been obtained regarding experimentally-induced synaptic plasticity in cortical cultures at the network level possibly due to overriding synaptic changes induced by global burst activity [14,15]. Furthermore, hypersynchronous activity can hinder effective signal processing of extracellular electrophysiological recordings [21,22] and provides a poorly suited driver for closed-loop neural animat (artificial animal) paradigms in which a culture is embodied using, for example, sensors and actuators of a mobile robot [23-25]. This latter point is of particular relevance as animat use has received considerable support as a platform for investigations of network level processing within a behavioural context without *a priori* knowledge of underlying cellular and/or molecular mechanisms [23,26-30]. Moreover, unrepresentative neuronal activity will hinder and confound the use of embodied cultured networks in the design of model neural systems and effective brain-computer interfaces for disabled human patients [31].

Global burst activity in cultures shares common features with neuronal activity exhibited during epileptic seizures *in vivo* and both states typically arise via excitatory and inhibitory synaptic imbalance [32]. We postulated that such activity could arise and/or be modulated by the release of ‘tonic’ endogenous neurotransmitter(s) within the culture environment (*e.g.* glutamate [33,34] or acetylcholine (ACh) [35]). In this regard, central muscarinic acetylcholine receptor (mAChR) activation causes *in vivo* seizure activity [36] and *in vitro* epileptiform activity in acute brain slices [37,38]; neuronal firing can be either increased or ablated by pharmacological manipulation of muscarinic ACh receptor (mAChR)-mediated postsynaptic increases in excitability and presynaptic inhibition of neurotransmitter release respectively [39,40]. In addition to modulating seizure-related excitability, mAChRs mediate a broad functional role *in vivo,* modulating plasticity, information flow, network communication and plateau potential generation [41] together with central pre- and postsynaptic nicotinic acetylcholine receptors (nAChRs) also influencing neuronal firing and transmitter release to modulate higher order functions in learning and memory [42].

Since all culture-based studies rely upon the tenet that some common features are shared with *in vivo* tissues, a means to modulate physiologically unrepresentative burst activity could support the growing need for better physiological and functional comparability. Thus, identification and manipulation of a postulated endogenous cholinergic system in cortical cultures represents an attractive target by which to achieve these ends. Here, using immunohistochemical, RT-qPCR and electrophysiological methods we show the presence of intrinsic cholinergic neurons and significant endogenous cholinergic ‘tone’ in cortical cultures grown on multi-electrode arrays (MEAs) and that tonic neuronal activation of both mAChRs and nAChRs affects both global excitability and burst event regularity in a culture age-dependent manner.

## Methods

### Cell culture

Timed pregnant Wistar-Kyoto dams (Charles River, Margate, Kent, UK) were sacrificed in accordance with the UK Animals (Scientific Procedures) Act 1986 by overdose with inhaled isoflurane (Merial Animal Health, Harlow, Essex, UK) at embryonic day 18. Embryos were then rapidly removed, decapitated and decerebrated on ice. Cortical sections from each foetus were removed under aseptic conditions and finely chopped in cooled phosphate-buffered saline (PBS; Lonza, Slough, Berkshire, UK). The tissue was then enzymatically dissociated in 0.04% trypsin EDTA (Invitrogen, Paisley, Renfrewshire, UK) at 37°C (NAPCO 6500 TC Incubator, Thomson Scientific, London, UK) before quenching with horse serum (Invitrogen) after 20 minutes. The clear phase of the resulting suspension was then pipetted off and made up to 10 ml with warm sterile PBS plus 20 mM glucose solution followed by 6-9 trituration passes and centrifugation at 800 rpm for 4 minutes at room temperature. The resulting cell pellet was resuspended in 12 ml of control media that consisted of Eagles minimum essential medium base (92.8% EMEM; Invitrogen) supplemented with gentamycin (0.1%; Invitrogen), 1 M glucose (1.5%; Sigma Aldrich, Poole, Dorset, UK), HrS (5%; Invitrogen) and L-glutamine (0.5%; Invitrogen). Viable cell density was determined by visual inspection on a haemocytometer by 0.4% trypan blue (Invitrogen) staining. Cultures were maintained by bi-weekly 50% media exchange and, after 7 days *in vitro* (DIV), media L-glutamine content was reduced to 0.25%.

### Seeding and restriction

Multi Electrode Arrays (see below) were soaked in an aqueous solution of 1% Terg-a-zyme (Alconox via Cole-Palmer, London, UK; ~20 minutes) before washing in 70% ethanol (Thermo-Fisher, Epsom, Surrey, UK), rinsing in ultrapure water and air drying before application of a sellotape inverse template equal in diameter to the MEA total electrode area (~1 mm^2^). MEAs were then autoclaved and coated by addition of 50 μl 0.1 mg/ml poly-d-lysine (Sigma Aldrich) on the electrode area for 20 minutes before rinsing in PBS (Invitrogen) and overnight sterilisation under ultraviolet light. Finally, MEAs were incubated with 1 ml of EMEM plus 10% foetal calf serum (Sigma Aldrich) for a minimum of 2 hours. This media was removed immediately prior to seeding and replaced with 1 ml of a 500,000 cells/ml suspension. Seeded cultures were allowed to settle for 30 minutes before template removal and addition of Potter rings [43]. When not under recording conditions (see below), cultures were maintained at 37°C, 5% CO_2_ in a humidified incubator.

### Developmental classification

Cultures were classified as either immature (DIV14-25) or mature (DIV37-61) on the basis of previously published developmental classifications (~DIV30; [44]). For brevity in text, the letters ‘m’ and ‘i’ are used to denote mature and immature respectively. The minority of cultures that failed to exhibit a mean global burst incidence of >0.25 Hz within 5 minutes of commencing control recordings were considered atypical and excluded from the present study.

### Immunohistochemistry

Cultures on cover slips were prepared and maintained in an identical manner to those seeded onto MEAs (see above) and using tissue from the same source embryos on three separate occasions. Cultures were fixed in freshly made 4% paraformaldehyde (pH 7.4, Sigma Aldrich) immediately prior to immunohistochemical staining. Cultures were washed in PBS three times between each of the following steps and all immunohistochemical reactions were conducted at room temperature. Non-specific binding was prevented by blocking with 10% normal goat serum (Invitrogen) in PBS and cells were permeabalised with 0.02% Triton TX-100 (Sigma Aldrich) for 5 minutes. Primary antibodies directed to ß-tubulin (1:500; Invitrogen; G7121), tyrosine kinase A (TrkA; 1:10000; Reichardt Lab, University of California; [45]), α-bungarotoxin-488 (100 nm; Invitrogen; B-14322), M1 (1:100 Millipore; AB5164) and M2 (1:200; Abcam; BA2805) were added for 1 hour before addition of the secondary antibodies, Red Alexa Fluor 568 (1:500; Invitrogen; A-11031) and Green Alexa Fluor 488 (1:500; Invitrogen; A-21206) for 30 minutes. Cell nuclei were stained using 10 mg/ml Hoechst blue 33342 (Invitrogen; H3570) for 5 seconds prior to cultures being mounted on standard glass slides (Thermo Fisher) using Vectashield (Vector Laboratories, Peterborough, Cambridgeshire, UK) and followed by storage at 4°C. Negative controls were produced by omission of primary or secondary antibodies. Positive controls were obtained from perfusion fixed medial septal slice tissue (50 μm coronal sections, +0.2 mm from Bregma) obtained from P > 40 Wistar-Kyoto rats and in which the primary antibody (1:500) was incubated at room temperature overnight. Fluorescence images were acquired using a Zeiss Axio Imager.A1 and an AxioCam MRm coupled with Axiovision software (Carl Zeiss MicroImaging, Welwyn Garden City, Hertfordshire, UK). Confocal fluorescence images were acquired using the Leica confocal laser unit (Leica, Milton Keynes, Buckinghamshire, UK) coupled to a Leica DM IRE2 microscope equipped with a Leica X63, 1.4 NA oil-immersion objective lens.

Ribonucleic acid (RNA) was extracted from cultures (grown, as described above, in T75 flasks) and septal sections (frozen on dry ice during dissection and subsequently homogenised) by addition of RNAbee (AMS Biotechnology Ltd, Abingdon, Oxfordshire, UK) for 5 minutes before addition of 200 μl chloroform (Sigma Aldrich) and centrifugation at 15,000 rpm (Biofuge 15R, Heraeus, Newport Pagnell, Buckinghamshire, UK). The clear phase was then removed, added to 500 μl isopropanol (Sigma Aldrich) and centrifuged again at 15,000 rpm. Finally, the pellet was dislodged, centrifuged in 1 ml of 75% ethanol at 8000 rpm for 4 minutes and air dried before resuspension in RNA resuspension buffer (2 M lithium chloride, 10 mM sodium acetate) at 65°C and stored at -80°C. Complementary deoxyribonucleic acid (DNA) was produced by reverse transcription of 2 μg of RNA in a total reaction volume of 40 μl containing: 8 μl MgCl_2_ (Promega, Southampton, Hampshire, UK), 4 μl 10x buffer (Promega), 4 μl deoxynucleoside triphosphates (dNTPS; GE Healthcare, Little Chalfont, Buckinghamshire, UK), 1 μl oligo-dT primer (Promega), and 0.5 μl reverse transcriptase (Promega) made up to volume with RNase and Dnase free water (Sigma Aldrich). Reactions were conducted at 42°C for 60 minutes followed by 95°C for 5 minutes. Specific oligonucleotide primers were designed to amplify 143 bases of TrkA (forward: TGATGCTGGCTTGTGCTTGCGCC, reverse CACATAGAGCTCCGTCAGGTTCCCGGC; accession number NM_021589) and 147 bases of choline acetyltransferase (forward: TGGTGTACAGCAGCGCTGGTTCGG, reverse: GCTCCTCCGGAAAAGAACAC CTCCCCC; accession number XM-001061520). Reverse transcriptase quantitative polymerase chain reactions (RT-qPCR) was carried out on a reaction volume of 14 μl that comprised 5 μl 1:50 cDNA diluted in TE buffer (Invitrogen), 1 μl forward primer, 1 μl reverse primer and 7 μl QuantiTect SYBR Green (Qiagen, Crawley, Sussex, UK) in a qPCR thermal cycler (Applied Biosystems, Paisley, Renfrewshire, UK) for 40 cycles. Products were confirmed via both melt curve analysis and visualisation by electrophoresis on a 3% agarose gel (Bioline, London, UK) with 1:10000 ethidium bromide (Sigma Aldrich). Β-actin primers (forward: ATCGTGGGCCGCCCTAGGCAC, reverse: TGGCCTTAGGGTTCAGAGGGGC; accession number NM031144) were used as a positive control alongside a ‘no sample’ negative control.

### Electrophysiological recording

All recordings were undertaken within a humidified 37°C, 5% CO_2_ incubator (NAPCO 6500 TC Incubator, Thomson Scientific) using MEAs sealed with Potter rings ( [43]; Scientifica, Brambleside, Sussex, UK). All electrical hardware was sealed in a custom box, open to the humidified environment only at the Potter ring membrane interface. Unit and multi-unit spontaneous neuronal spike firing events were electrophysiologically recorded via ‘8 × 8’ MEAs of 59 planar electrodes (30 μm diameter; 200 μm inter-electrode spacing; Multi Channel Systems, Reutlingen, Germany) housed in a MEA1060BC headstage (Multi Channel Systems). The stability of the recording environment was periodically assessed by examining the mean array-wide firing rate (bin size: 5 mins) over a 72 hour recording period in addition to continuous monitoring of incubator humidity and temperature. Movement-induced changes in activity [17] were mitigated against by observing a 10 minute waiting period following MEA insertion in the headstage or drug application, but before beginning data acquisition. Signals were amplified (1100× gain), band-pass filtered (10-3200 Hz) and recorded as raw data streams and spike cut-outs (Limada threshold: 5.5) in parallel at 25 kHz using MEABench [44]. Following a given experiment, drug was washed off by immediately replacing 100% of the media followed by least two 50% media changes during the following seven days. A minimum waiting period of seven days between successive experiments employing a given culture was established by comparison of the array-wide spike rate in control conditions prior to the application of drug with the same measure derived from a recording made under the same conditions seven days later; no significant difference was found between these pairs of recordings (n = 5; P > 0.5).

Cholinergic agents used in this study were oxotremorine methiodide (OXO-M), atropine (sulfate monohydrate), and mecamylamine (nicotine hydrogen tartrate; MEC), each of which were obtained from Sigma Aldrich (UK). Since mAChR activation can cause neuronal depolarization and the appearance of slow post-burst afterdepolarizing potentials in cortical neurons (sADPs: [46]) and the same phenomenon can also be induced by cortical metabotropic glutamate receptor (mGluR) activation [46], the group I/II mGluR antagonist (S)-α-methyl-4-carboxyphenylglycine (MCPG: 1 mM) was applied in the absence and presence of the mAChR agonist, OXO-M (10 μM) to prevent possible mGluR-mediated postsynaptic events contributing to activity changes in states of high mAChR activation [47]. No significant difference between any measured parameter relative to control was found in the presence of MCPG alone or between OXO-M responses obtained in the presence and absence of MCPG. Where multiple antagonists were sequentially applied, the effect of application sequence was examined, but no statistically significant differences between any measures were found.

### Signal post-processing and analysis

All signal processing was performed using in-house MATLAB (v2007b; The Mathworks) tools. Global burst event identification was performed using the MATLAB-based SIMMUX [44] algorithm, adapted to provide reliable burst identification under the conditions employed in the present study, particularly OXO-M-treated states where high levels of tonic firing were evident. The criteria used for global burst identification were ≥4 spikes within 100 ms on ≥4 individual channels which overlapped within in a 250 ms window (see [48] for a review of burst detection methods). Positive-going spikes of ≤50 μV amplitude were taken as representative of the rectification phase of a previous negative-going spike and so were not considered as events in any recordings [49]. The definitions of analysis measures used to quantify changes to spike firing features are shown in Table 1. With the exception of MEC in immature cultures (n = 5), six (MEA) replicates for the different drug states in mature and immature cultures were used.

**Table 1:** Formal definitions of the measures employed by the current study and their abbreviations used in accompanying figures

**Measure name**	**Abbreviated label used in figures**	**Formal definition**
Network spike rate	**Not applicable**	Total number of spike events occurring on all channels within a 4 ms bin.
Network spike profile	**Not applicable**	Network spike rate *vs* time during global burst events. T_0_ is defined by the time at which the bin containing the maximum rate occurred and is based on the Simmux algorithm [17].
Spikes per channel during global bursts	C-spikes	Number of spike events per channel during a global burst event.
Spikes on all channels during global bursts	G-spikes	The sum of spike events occurring on all channels during a global burst event.
Active channels during global bursts	C-active	Number of channels showing spiking activity during a global burst event when single channel burst criteria are met; see Methods.
Burst duration (channel)	C-duration	Time between first and last spike events on a channel during a global burst event.
Burst duration (global)	G-duration	Time between first and last spike events on all participating channels during a global burst event.
In-burst ISI	ISI-burst	Mean interspike interval derived from all events occurring on all channels during a global burst event.
Overall ISI	ISI-all	Mean interspike interval derived from all events occurring on all channels during a given recording.
Proportion of spikes occurring in bursts	SIB	‘Spikes in bursts’: spike events occurring within global burst events as a proportion of the total number of spike events occurring during a recording.
Inter-burst interval	IBI	‘Inter-burst interval’: time between the last spike of a preceding burst event and the first spike of the next burst event.

Representative traces were produced from raw data files using custom MATLAB functions. Due to inherent culture-to-culture variability in basal neuronal activity, measures of drug-induced effects were normalised by calculating the difference between a measure in a drug-treated state from the control state on the same culture and expressed as a percentage of the control value (i.e. percentage change from starting control values). Histograms for given measures (Table 1) were constructed in a similar manner by applying the same process to derive values for each time bin. This approach, as opposed to representations solely by means, was employed to produce visually clear plots. All data are presented as mean ± SEM. Differences between control and multiple drug states for measures listed in Table 1 were assessed using a Wilcoxon signed rank test and the family-wise error rate controlled by taking *P* ≤ 0.05/4. A two-way ANOVA was employed to establish differences in burst profile between control and drug-treated states and significant differences accepted at P ≤ 0.05. A Student’s *t*-test was used to assess drug effects on non-normalised means describing spike firing measures.

### Preparation of drug stocks

All drugs were dissolved in sterile filtered dH_2_O, divided into 100 μl aliquots and stored at -20°C until use. Drug stocks were thawed before application by pipette (50 μl volume) directly to the MEA recording chamber to achieve the desired final bath concentration. Application of vehicle (50 μl dH_2_O; n = 6; data not shown) produced no significant changes to any of the activity measures employed. In all cases following drug application, an equilibration period of 10 minutes was observed prior to electrophysiological recording.

## Results

### Developmental differences in spontaneous neuronal activity

Primary cortical cultures display complex spontaneous spike firing patterns with considerable developmental variation [17] that is subserved by a variety of neuronal cell types including principal glutamatergic neurons, GABAergic interneurons and glial types [14,17]. Consequently, and prior to undertaking investigations of cholinergic responsiveness, we developmentally characterised spontaneous neuronal activity exhibited by these cultures (Tables 1 &2).

**Table 2:** Electrophysiological measures of spiking and bursting activity from mature and immature cortical cultures

**Age**	**Spikes per channel during global bursts**		**Spikes on all channels during global burst**		**Active channels during global bursts**		**Burst duration (channel; ms)**		**Burst duration (global; ms)**	
	**Range**	**Mean ± SEM**	**Range**	**Mean ± SEM**	**Range**	**Mean ± SEM**	**Range**	**Mean ± SEM**	**Range**	**Mean ± SEM**
**i**	9-23	13 ± 1^*^	70-592	264 ± 43	7-38	19 ± 2^*^	20-80	50 ± 5	80-190	140 ± 8^*^
**m**	6-20	11 ± 1	35-374	140 ± 22	5-20	12 ± 1	20-50	40 ± 3	40-160	100 ± 1
	**In-burst ISI (ms)**		**Overall ISI (ms)**		**Proportion of spikes in bursts (%)**		**Inter-burst interval (s)**			
	**Range**	**Mean ± SEM**	**Range**	**Mean ± SEM**	**Range**	**Mean ± SEM**	**Range**	**Mean ± SEM**		
**i**	2-9	4.6 ± 0.5	13-61	33 ± 4	28-93	56 ± 6.5	1.9-30.1	7.3 ± 2.0		
**m**	2-6	4 ± 0.3	22-79	44 ± 4	26-91	58 ± 4.8	0.9-27.2	5.1 ± 1.5		

Data are presented as minima and maxima ranges and means ± SEM. ‘m’ and ‘i’ represent mature and immature cultures respectively (see Methods). * indicates significant differences (P ≤ 0.05) between mature and immature cultures for a given measure.

**Figure 1:**
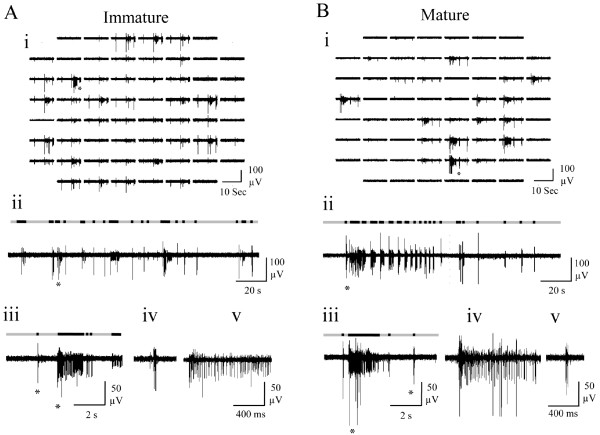
**Representative spontaneous neuronal activity exhibited by (A) immature and (B) mature cortical cultures recorded via MEA under control conditions.** (i) Array-wide neuronal activity. (ii) An extended period of spontaneous neuronal activity (160 s) recorded from a single channel. (iii) An expanded single channel trace obtained from the period indicated by a star in ii. (iv) Expanded burst events from the left and (v) right star in iii. Superimposed black sections within grey bars represent bursts identified within the raw traces shown by the adapted SIMMUX algorithm. Note that neural activity was generally more evident across the array channels in the immature culture.

Both immature (‘i’; Figure 1Ai) and mature (‘m’; Figure 1Bi) cultures spontaneously exhibited variable duration bursts, containing ~50% of spiking events observed and separated by variable inter-burst intervals during which sparse tonic firing was evident (Figures 1Aii and 1Bii). Bursts typically consisted of an initial large amplitude compound spike followed by a variable duration train of mixed amplitude events. Immature cultures exhibited spontaneous activity on a significantly larger number of channels, generated significantly more spikes during significantly longer global bursts consistent with a higher level of overall network excitability (Table 2). No other significant differences between mature and immature cultures were found, although it is notable that the considerable variability observed between cultures (‘Range’ in Table 2) would hinder such comparisons. Thus, culture responses to pharmacological treatment reported hereafter are expressed as percentage changes from starting control values. However, these data confirm that spontaneous neuronal activity exhibited by the cultures employed in the present study is comparable to that previously reported [16,17,50].

### mAChR agonist-induced changes in spontaneous activity

**Figure 2:**
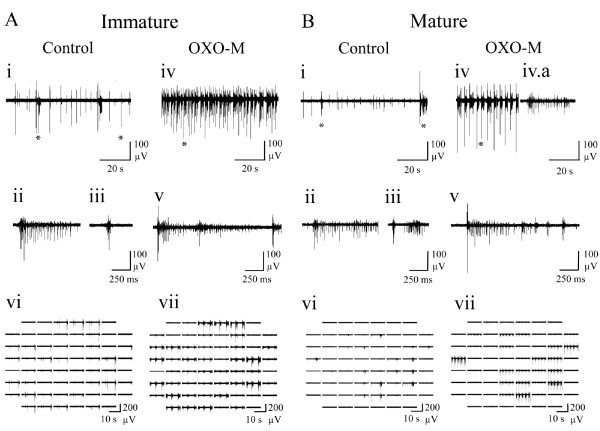
**Representative spontaneous neuronal activity exhibited by (A) immature and (B) mature cortical cultures recorded via MEA in control and following application of the mAChR agonist, OXO-M (10 μM). **(i) 100 s of spontaneous neuronal activity occurring on a single channel, where the left and right asterisks indicate periods later expanded to show a long (ii) and short burst (iii) respectively. (iv) ~50 s of activity following application of OXO-M, showing occurrence of bursting and (iv.a) asynchronous mixed tonic and bursting activity types. (v) Expanded period indicated by asterisk in iv. (vi) Array-wide neuronal activity in control conditions and (vii) following addition of OXO-M. Note the appearance of burst events on several channels in OXO-M, in both mature and immature cultures.

Spontaneous neuronal firing by mature (DIV 40-57) primary cortical cultures on MEAs has been reported to desynchronize in response to application of the non-hydrolysable mAChR agonist, carbachol [13,51]. In order to confirm comparable responsiveness in the cultures employed here, to further elucidate the contribution of mAChR-mediated modulation to spontaneous neuronal activity and assess possible developmental changes in cholinergic responsiveness, the effects of the non-hydrolysable mAChR agonist, OXO-M were first assessed (Figure 2).

**Figure 3:**
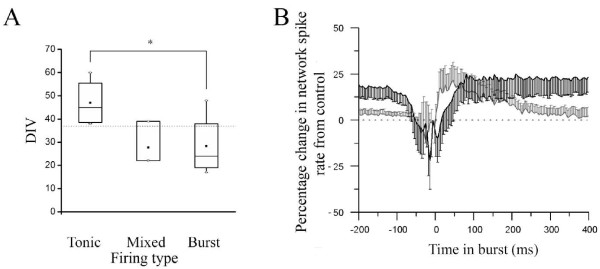
**Developmental differences in the effect of mAChR activation on spontaneous neuronal activity recorded from cortical cultures via MEA.** (**A**) The relationship between culture developmental stage (DIV) and firing type following mAChR activation by 10 μM OXO-M. Outer box bottom = 25th percentile, top = 75th percentile, error bars bottom = 5th percentile, top = 95th percentile, central line = median, central square = mean. **B**) Network spike profile (see Table 1) for immature (grey) and mature (black) cultures presented as normalised change to network spiking rate *vs* control following mAChR activation. t_0_ = time bin at which maximum firing rate was detected.

In both immature (Figure 2Ai-vii) and mature (Figure 2Bi-vii) cultures, OXO-M (10 μM) caused changes in spontaneous spike firing characterised by a transition to highly organised and reproducible global bursts, continuous tonic firing with transient increases in firing frequency or a mixture of these two activity types. Analysis of the culture ages at which these three activity types manifested revealed that OXO-M caused tonic firing in mature cultures but burst firing in immature cultures (Figure 3A). Whilst OXO-M elicited classical bursting activity separated by periods of quiescence during which little or no tonic activity was evident (Figure 2Aiv) from immature cultures, it is notable that the predominantly asynchronous tonic firing it caused in mature cultures also included transient, multiple channel, firing frequency increases at intervals consistent with burst event incidence seen under control conditions (Figure 2Bi). Thus, for the comparative analysis undertaken here, such increases in mature cultures are categorised as burst events.

**Figure 4:**
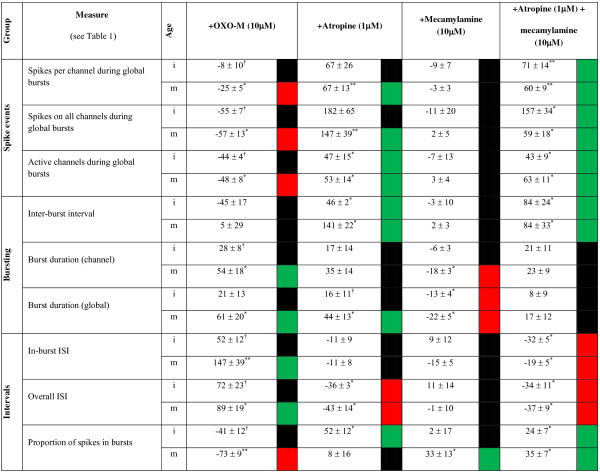
**Steady-state acetylcholine receptor ligand-induced changes to spiking and bursting measures expressed as percentage change from starting control values.** * = P ≤ 0.05, ** = P ≤ 0.01, *** = P ≤ 0.001 and † = a statistically strong trend (P ≤ 0.1) for comparisons *vs* starting controls. “red square symbol”, “green square symbol” and ■ summarise significant (P ≤ 0.05) increases, decreases or no change (P > 0.05) *vs* control respectively.

Interestingly, quantification of the effects of OXO-M upon the neuronal firing measures employed revealed changes in immature and mature cultures that were consistent in their direction but not extent. Here, in mature cultures, OXO-M significantly increased ISI (overall and in-burst), reduced the proportion of spiking activity occurring in bursts and the number of involved channels despite concomitant increases in channel and global burst durations (Figure 4).

Notably, whilst OXO-M affected the activity measures obtained from immature cultures in the same direction (Figure 4), only strong trends to differ from control (*P* ≤ 0.1) were found which suggest an attenuated mAChR-mediated response in immature (*c.f.* mature) cultures. Such similarities in the direction of OXO-M-induced changes could initially appear contradictory when set beside the gross developmental differences between bursting (immature) and tonic firing (mature) activities observed (Figure 3A). However, the underlying reasons for this can be most clearly visualised via network spike profiles [16,17,51] (Figure 3B; see Methods) since OXO-M significantly (P ≤ 0.05) increased mean network spike rate during non-bursting periods in mature, but not immature cultures. This specific developmental difference which occurred outside of burst events notwithstanding, OXO-M-induced changes in activity during bursts did not differ from control for either immature or mature cultures (Figure 3B). Finally, contrary to the typically attenuated muscarinic responsiveness seen in immature cultures, OXO-M significantly increased spike amplitude when compared with control conditions in immature (P ≤ 0.05) but not mature cultures (m: 0 ± 2%, i: 10 ± 3%). However, care should be taken with interpretation of such changes since the diverse neuronal types (each with different intrinsic firing properties) present in cultures make drug-induced changes to mixed population means not amenable to detailed insight. Thus, whilst the above result may represent a greater propensity for postsynaptic mAChR modulation of excitability in immature cultures [37] further study using single cell recording and/or spike sorting approaches would be required to fully elucidate the importance of this finding. Overall, these data not only confirm the presence of functional mAChRs capable of significant modulation of neuronal excitability consistent with that previously characterised in *in vitro* and *in vivo* CNS preparations [37,38,52] but also reveal interesting developmental changes to such responsiveness.

### Endogenous cholinergic tone

Whilst the responses to OXO-M demonstrate the presence of functional mAChRs and concur with previously presented results [13,51], the presence of sufficient endogenous ACh to modulate basal spontaneous neuronal activity in cortical cultures has not been demonstrated. Consequently, we employed immunohistochemical and reverse transcription-quantitative polymerase chain reaction (RT-qPCR) methods to investigate the presence of ACh-producing and AChR-expressing cells in our cultures.

**Figure 5:**
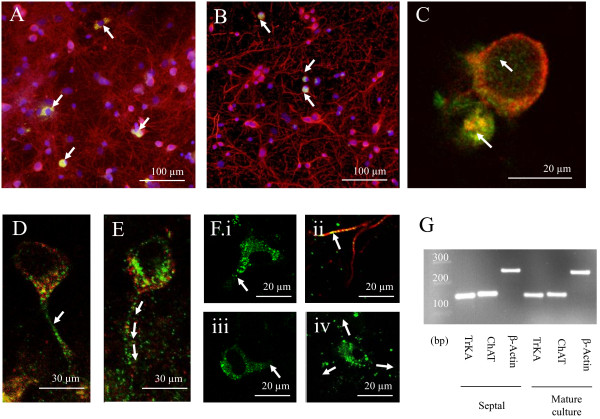
**Immunohistochemical and RT PCR studies confirm the presence of TrkA in mature and immature cortical cultures alongside α7-containing nAChRs and m2 mAChRs.** Fluorescence image of TrkA-immunoreactivity (green; see arrows) co-stained with neuron-specific ß-tubulin (red) and DNA nuclear stain Hoechst 33342 (blue) in mature (**A**) and immature (**B**) cultures. (**C**) Confocal image of TrKA localised to both the cytosol (upper arrow) and on the surface of a different cell located at a lower level in the Z-stack (lower arrow) in a mature culture. (**D&E**) Co-localisation of m2 mAChR (red) and m1 mAChRs (green) showing relatively higher levels of m1 mAChR expression along the apical dendrite (arrows) and general expression throughout the neuropil in a mature culture. (**F** i-iv) Confocal images of α7-containing nAChR positive cells (green) demonstrating the high level of α7-containing nAChR expression both on the soma and apical dendrite but limited punctate expression throughout the dendritic tree (Fiv arrows) and labelled with beta tubulin in (ii) in a mature culture. (**G**) Electrophoresis of RT PCR products shows corresponding bands at 143 (TrkA) and 147 (ChAT) and 244 (ß-actin) in both mature cultures and rat medial septum tissue positive control.

Morphologically diverse cells from both immature and mature cultures showed robust immunoreactivity for the TrkA antibody (Figure 5A) on the cell surface and within the cytosol (Figure 5Ai; ~95% of TrkA-expressing cells co-express choline acetyltransferase (ChAT) [45]). Cultures also expressed M1 and M2 mAChRs as well as α7-containing nAChRs (Figure 5B-F) where strong M1 and M2 mAChR expression was found on somata surfaces, apical dendrites and throughout the dendritic tree for a number of morphologically distinct cell types (Figure 5B-E). In contrast, α7 containing nAChRs were strongly expressed at somata surfaces and the initial apical dendrite for a number of cellular morphologies (Figure 5Fi-iv) but showed only limited punctate expression in dendritic trees and within the neuropil (Figure 5Eiv; arrows). RNA extracted from cultures contained both TrkA and ChAT mRNA (Figure 5G) although no significant differences in expression levels were found between immature and mature cultures (TrkA RT-PCR cycle times m: 11.1 ± 1.1; i: 9.4 ± 0.5, ChAT RT-PCR cycle times m: 12.3 ± 0.6; i: 12.4 ± 0.2; P > 0.05 for m *vs* i for TrkA and ChAT). These results support the presence of both ACh-producing cells and differentially-expressed muscarinic and nicotinic AChRs within the neuronal population comprising these cortical cultures.

### mAChR-mediated effects of endogenous ACh upon spontaneous spike firing activity

Given that our immunohistochemical and RT-qPCR results support the presence of intrinsic ACh-producing neurons in the cultures, we next investigated the effects of the mAChR-selective antagonist atropine alone (1 μM) upon spike firing measures, to pharmacologically establish whether endogenously-released ACh concentrations had attained functionally relevant levels that could affect spontaneous neuronal activity.

**Figure 6:**
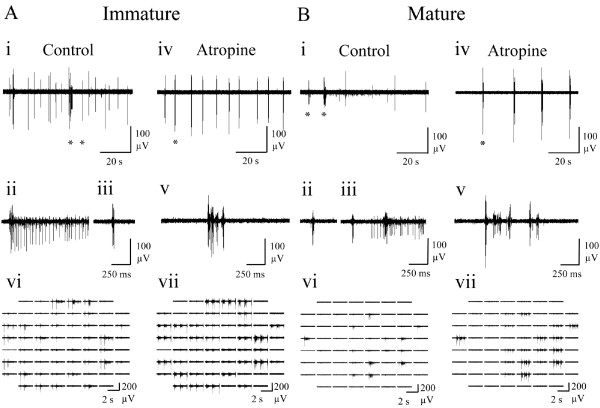
**Representative spontaneous neuronal activity exhibited by (A) immature and (B) mature cortical cultures following the addition of the mAChR antagonist, atropine (1 μM) and recorded via MEA. **(i) Spontaneous neuronal activity (100 s) recorded from a single channel. Left and right periods highlighted by an asterisk are expanded to show a long (ii) and short bursts (iii) respectively. (iv) Spontaneous neuronal activity (100 s) following atropine application where the period indicated by an asterisk is shown expanded in (v). (vi) Array-wide neuronal activity in control conditions and (vii) following addition of atropine. Atropine converted irregular firing in control conditions into synchronised burst firing for both mature and immature cultures.

**Figure 7:**
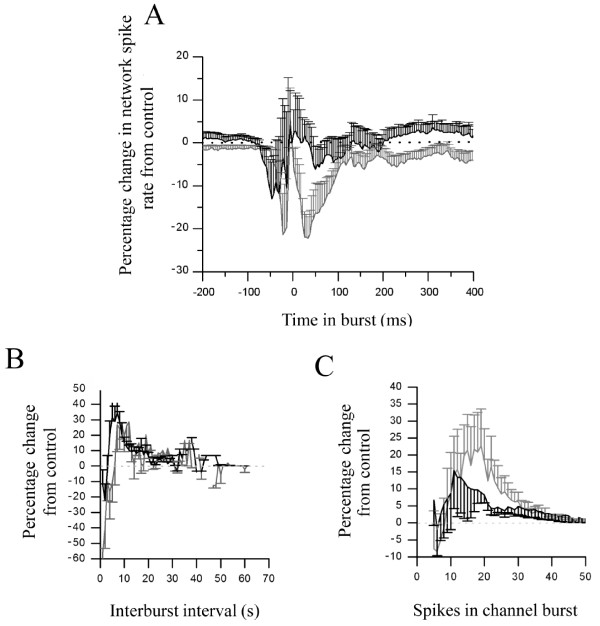
**mAChR blockade by atropine (1 μM) modulates spontaneous neuronal activity in cortical cultures recorded via MEA.** (**A**) Network spike profile (see Table 1) for immature (grey) and mature (black) cultures presented as normalised change from control following mAChR blockade. (**B**) The effect of atropine upon the distribution of interburst intervals in immature (grey) and mature (black) cortical cultures. (**C**) The effect of atropine upon the number of spikes occurring on individual channels during bursting.

In both immature (Figure 6A) and mature (Figure 6B) cultures, atropine produced a clear change from the previously described heterogenous (mixed tonic and bursting) spontaneous activity in control conditions to regularly spaced, spontaneous burst events of uniform duration and a concurrent reduction of asynchronous tonic activity. Interestingly and like OXO-M, atropine significantly increased spike amplitude when compared with control conditions in immature (P ≤ 0.05) but not mature cultures (m: 3 ± 4%, i: 50 ± 21%). For the previously noted reasons this finding was not amenable to further interpretation but could arise from reduced use-dependent accommodation of spike amplitude [37] consistent with the reduced spike rates reported hereafter. The developmental difference in response to mAChR blockade is best illustrated by the network spike profile where significant changes to the temporal distribution of in-burst spiking activity in immature (P ≤ 0.05), but not mature cultures were seen (Figure 7A). Notably, whilst atropine reduced in-burst spike rate in immature cultures compared to control conditions (Figure 7A), the paucity of tonic spiking between bursts is not only consistent with the significantly increased proportion of spiking events associated with bursts but also the significant reduction in overall ISI (Figure 4).

The regularity of bursting activity induced by mAChR blockade is further emphasised by significant atropine-induced changes to IBI distribution for both culture ages (P ≤ 0.05) where decreases in shorter IBIs (<5s; Figure 7B) in immature (but not mature) cultures were seen with concomitant increases in longer IBIs (~10s; Figure 7B) in both immature and mature cultures, manifesting as significant increases in mean IBI in both cases (Figure 4). Whilst mAChR blockade also significantly (P ≤ 0.05) affected the distribution of the number of spikes occurring in bursts on single channels in both immature and mature cultures (Figure 7C), the variability of responsiveness seen in immature cultures revealed a significant increase in both the overall number of spikes occurring within bursts and burst duration only in mature cultures (Figure 4). Finally, it is notable that mAChR blockade significantly increased the number of channels participating in global bursts in both immature and mature cultures (Figure 4).

In concert with the immunohistochemical and RT-qPCR results also presented, these effects of mAChR blockade support the presence of functionally significant (muscarinic) cholinergic tone, capable of modulating spontaneous neuronal activity in this preparation. In summary, mAChR blockade in both mature and immature cultures, induced a significant shift towards increasingly synchronised and invariant activity which manifested as highly regular burst events comprising more action potentials than present in control conditions.

### nAChR-mediated effects of endogenous ACh upon spontaneous spike firing activity

**Figure 8:**
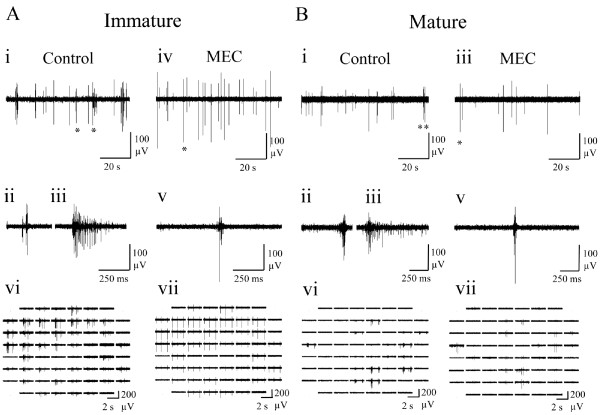
**Representative spontaneous neuronal activity exhibited by (A) immature and (B) mature cortical cultures following application of the nAChR antagonist, mecamylamine (10 μM; MEC). **(i) Spontaneous neuronal activity (100 s) on a single channel where left and right asterisks indicate typical short and long bursts that are expanded in (ii) and (iii) respectively. (iv) Spontaneous activity (100 s) following mecamylamine application where the period indicated by an asterisk is shown expanded in (v). (vi) Array-wide neuronal activity in control conditions and (vii) following mecamylamine application. Note the limited effect of nAChR blockade (*c.f.* mAChR blockade; Figures 6 &7) such that only burst duration was significantly affected by mecamylamine (Figure 4).

The effects of the mAChR agonist OXO-M, confirmed the presence of functional mAChRs and immunohistochemical, RT-qPCR and mAChR pharmacological blockade supported the presence of sufficient endogenous cholinergic tone to modulate basal spontaneous activity via mAChRs. It was also of interest to examine whether functional nAChRs were also present in these cultures and whether their tonic activation by endogenous ACh could affect spontaneous spike firing. However, unlike the significant changes from control observed following mAChR blockade for the majority of measures employed (Figures 6 &7; Figure 4), the effects of applying the nAChR-selective antagonist MEC (10 μM) were more limited (Figure 8). Thus, nAChR blockade only caused a significant decrease in burst duration for both immature and mature cultures and an increase in the proportion of spikes occurring in bursts for mature cultures only (Figure 4). Unlike, OXO-M and atropine, MEC had no significant effect upon spike amplitude when compared with control conditions in either immature or mature cultures (m: 3 ± 2%, i: 1 ± 1%; P > 0.05 vs control in each case).

### Spontaneous neuronal activity in the absence of cholinergic modulation

**Figure 9:**
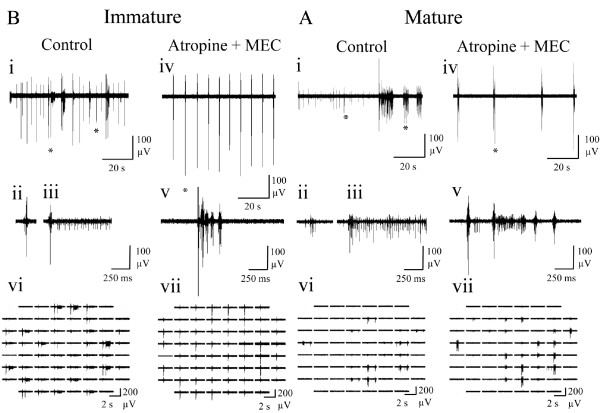
**Representative spontaneous neuronal activity recorded via MEA and exhibited by (A) mature and (B) immature cortical cultures following the blockade of all endogenous ACh effects by addition of atropine (1 μM) plus mecamylamine (10 μM; MEC). **(i) Spontaneous neuronal activity (100 s) recorded in control conditions on a single channel where left and right asterisks indicate typical short and long bursts expanded in (ii) and (iii) respectively. (iv) Spontaneous neuronal activity (100 s) following atropine plus MEC application where left and right asterisks indicate typical short and long bursts expanded in (v). (vi) Array-wide spontaneous neuronal activity in control conditions and (vii) following addition of atropine plus MEC. Note the similarity between atropine plus MEC treatment and atropine treatment alone (Figure 6).

Finally, to assess total ‘tonic’ cholinergic influences on spontaneous firing behaviour, all cultures were also treated with a combination of atropine (1 μM) plus MEC (10 μM) in order to block activation of both mAChRs and nAChRs (Figure 9). The results obtained were very consistent in direction and extent with those seen following the blockade of mAChRs alone (Figure 4; Figures 6 &7) which is perhaps unsurprising given the limited effects caused by nAChR blockade alone (Figure 4 & Figure 8). The only notable differences in significant effects upon burst measures following mAChR plus nAChR blockade that were not seen following mAChR blockade alone were an increased proportion of spikes occurring within bursts in immature cultures and a decrease in in-burst ISI for both immature and mature cultures, although the direction of both changes were consistent with those seen following mAChR blockade alone (Figure 4).

**Figure 10:**
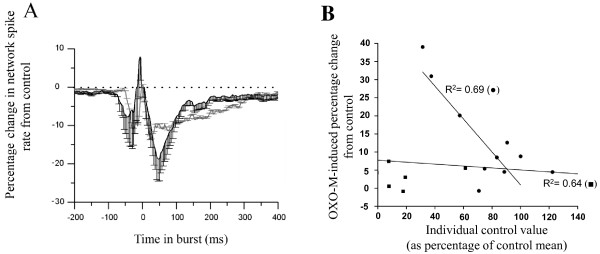
**The effect of mAChR and nAChR blockade on the network spike profile and the correlation between starting state and mAChR-mediated response.** (**A**) Network spike profile (see Table 1) for immature (grey) and mature (black) cultures presented as normalised change *vs.* control following combined mAChR plus nAChR blockade by atropine (1 μM) and mecamylamine (10 μM) respectively. (**B**) The relationship between starting state and extent of change following mAChR activation by OXO-M (10 μM) in mature cultures where ■  = global burst duration and ●  = single channel burst duration.

The network spike profile obtained following mAChR plus nAChR blockade (Figure 10A) again revealed changes in both immature and mature cultures that were comparable to those seen mAChR blockade alone (*c.f.* Figure 7) although, notably, unlike the latter condition, significant effects (P ≤ 0.05) upon the temporal distribution of spike firing were only seen in immature cultures, most likely due to the reduced variability of in-burst spike firing (Figure 7A vs Figure 10A).

Consistent with the dominance of muscarinic effects upon spike firing measures, mAChR plus nAChR blockade significantly increased spike amplitude in immature (P ≤ 0.05) but not mature cultures to an extent comparable to that seen following mAChR blockade alone (m: 3 ± 4%, i: 49 ± 21%).

Overall, these findings suggest a dominance of mAChR-mediated influences across the cellular population as a whole with nAChR-mediated modulation being less prevalent and possibly circuit specific.

### Basal ACh levels affect agonist responsiveness

Interestingly, the consequences of applied mAChR agonist treatment upon some burst measures appeared to be related to the starting (control) amplitude of the measure for a given trial (Figure 10B) such that two notable examples of negative correlation were apparent in mature cultures. Cultures exhibiting lower starting values for a given measure typically exhibited the most substantial changes following mAChR activation, whilst those cultures with higher starting values exhibited little change following mAChR agonist application. This is suggestive of temporal and inter-culture variation in endogenous ACh-mediated activation of mAChRs that is not only consistent with intact tissue and/or *in vivo* ACh level fluctuations but also could be experimentally exploited. In addition, this relationship was found exclusively in mature cultures and therefore adds support to the previously described attenuated immature mAChR response.

## Discussion

This study has demonstrated, for the first time, the presence of significant, culture age-dependent, endogenous cholinergic tone in cortical cultures of embryonic origin that orchestrates a neuromodulatory role largely consistent with that reported in intact cortical networks *in vitro*[37,38,40,52-54] and *in vivo*[55]. This finding adds credence to the previously identified conservation of underlying cellular and network mechanisms in such preparations [1,56] despite the absence of behaviourally relevant, environmental and genetic regulation. In summary, endogenous ACh activating mAChRs broadly modulated excitability and consequently determined spatiotemporal variability of activity whilst endogenous ACh effects upon nAChRs manifested as a more restricted, subtle, but significant modulation of excitability.

### *mAChR-mediated responsiveness*

Whilst the spontaneous activity types exhibited by both immature and mature cultures in control conditions were both largely similar and consistent with previous reports [16,17,50], responsiveness developmentally diverged upon pharmacological modulation of cholinergic receptors.

Agonist-induced mAChR activation with OXO-M, altered control firing activity characterised primarily by global burst events interspersed with low levels of tonic activity, to either persistent asynchronous firing coupled with transient global firing frequency increases in mature cultures, or predominantly global bursting activity in immature cultures; these changes were consistent with effects of the mAChR agonist, carbachol, on cortical/MEA cultures reported by Tateno *et al.*[13,51] and so importantly, support commonalities between these preparations.

The similar nature of these changes in activity to those seen *in vivo* and in acute brain slices *in vitro* in response to mAChR activation, are consistent with similar underlying ionic mechanisms; i.e. predominant increases in postsynaptic excitability via modulation of membrane K^+^ currents [57-59] and non-selective cationic conductances [60] as well as generation of graded persistent activity through induction of slow post-stimulus afterdepolarizing potentials [46,61]. Whilst the relationship between increased postsynaptic excitability and the appearance of asynchronous activity could initially appear contradictory, it could arise from a larger numbers of cells simultaneously entering a refractory period at any given time and thus preventing the cascade of activity through the network [62,63].

In contrast to the persistent asynchronous activity observed in mature cultures following mAChR activation, immature cultures displayed periods of quiescence between bursts, unlike the sparse levels of tonic activity seen during control recordings. Interestingly, a comparable age-dependent differential consequence has been reported in acute piriform cortical brain slice neurons, where mAChR activation by OXO-M caused increases in spontaneous tonic firing activity in adult (>40 days post-natal) slices but elicited spontaneous epileptiform activity in immature (14-28 day post-natal) slices, characterised by clearly defined periods of high frequency bursting separated by periods of quiescence [37,40]. Although we detected both M1 and M2 mAChRs in our cultures, it is not possible to assign pharmacological specificity to the effects on firing in mature *vs* immature neurons described here as this requires further investigation using subtype selective mAChR antagonists.

### mAChR-mediated influence of endogenous ACh upon spontaneous spike firing activity

It is most likely that the presence of significant endogenous ACh release in cortical cultures has not previously been considered due to the exclusion of tissue containing cholinergic cell bodies (*e.g.* medial septum) during the preparation of cortical/forebrain tissue for culture. However, several studies support mechanisms from which physiologically significant levels of neuromodulatory transmitters can arise in cortical cultures which include environmentally-dependent progenitor differentiation into neuromodulatory phenotypes [64,65] and the presence of intrinsic cholinergic cells and synapses within rat cerebral cortex itself [66]. Here, we confirmed the presence of ChAT and TrkA mRNA and the distribution of TrkA- expressing cells [45] along with the presence of M1 and M2 mAChRs and α7 nAChRs.

Blockade of the effects of mAChR-mediated endogenous cholinergic tone by atropine significantly increased excitability within global bursts and their regularity, suggesting that high tonic ACh levels could underlie the observed variability within the temporal structures of both burst and tonic spontaneous neuronal activity (Figures 3*vs*6). We thus propose that the regularity of spontaneous neuronal activity exhibited by cultures in the absence of mAChR-mediated contributions could be attributed to comparable mechanisms as those *in vivo*, although tempered by the highly connected nature of cultures. In addition, increased synchronous neuronal activation following mAChR blockade could arise from the removal of tonic mAChR-mediated *pre*synaptic inhibition of excitatory neurotransmitter release [40,67]. Accordingly, such presynaptic disinhibition would result in the entraining of the majority of units during a global burst and so concomitantly synchronise the refractory periods of involved units which could underlie the reduced variability in burst timing [55,68]. It is notable that a previous investigation of mAChR agonist effects upon cortical cultures [13,51] reported that atropine (10 μM) application in the continued presence of the mAChR agonist muscarine (20 μM) inconsistently abolished spontaneous activity, although the application of atropine alone did not. This is in contrast to the results presented here where the effects of mAChR agonist plus antagonist and the application of antagonist alone did not significantly differ. It is uncertain whether this difference between studies is a result of differences between the muscarinic agonists used or the high concentration of atropine they applied, exerting non-specific effects (10 μM; [69]). Alternatively, the variability in responsiveness reported by Tateno *et al.*[13,51] could suggest a potentially high degree of variability in presence or level of endogenous cholinergic ‘tone’ under the conditions employed.

### nAChR-mediated responsiveness

The significant decreases observed in both channel and global burst durations, coupled with a concomitant reduction in the number of spikes occurring outside burst events in the presence of the nAChR antagonist MEC, suggests a predominantly excitatory role for nAChRs in these preparations. The more limited influence of nAChRs, in comparison to the more extensive mAChR network influence, suggests that the effects of nAChR activation may be restricted to a subset of neural circuits as found *in vivo*[70]. However, the combination of network mapping using high spatial resolution recording techniques such as calcium imaging, or high resolution MEAs, would be required to confirm this.

Finally, the distribution of α7-containing nAChRs appeared relatively limited throughout the dendritic tree relative to the ultrastructural location of nAChRs *in vivo*[71]. However, as nAChRs are preferentially expressed on neurons processing afferent sensory information *in vivo*[72,73], the differences seen in culture may be a product of the lack of afferent sensory input [74] and would make a comparison of nAChR levels and distribution following artificially introduced re-afferentation [75] of considerable interest. It is also worth noting that acetylcholinesterase (AChE) is expressed in primary cortical cultures [76,77]; a comparison of the effects of atropine and MEC described above, with those of an AChE inhibitor such as neostigmine would also be worthwhile (*c.f.*[38]).

### Implications for the cortical cell culture model and their use in animat paradigms

As previously described, cultures exhibiting mixed (tonic and bursting) activity would be more representative of activity observed in intact cortex and therefore provide a more suitable substrate not only for conventional studies (see Background) but especially as a driver for neural animat paradigms. The results presented here suggest that the cholinergic system is an attractive target for such an improvement in addition to providing opportunities to influence mAChR- and/or nAChR-mediated effects upon synaptic plasticity [78-81]; a core feature in such studies that has thus far been unreliable in this preparation type [14]. Moreover, if endogenous ACh levels are indeed temporally variable (Figure 9B) as our results suggest, such intrinsic variation also presents an attractive feature for exploitation by animat implementations since periods of ‘attention’ (during training stimulation) and ‘consolidation’ (after training stimulation) are necessary and so could be conducted during periods of ‘high’ and ‘low’ cholinergic tone respectively.

## Conclusions

In conclusion, we propose that the presence of an intact cholinergic system which exerts tonic effects upon neuronal activity in this preparation make it well suited to exploitation in animat and related paradigms by offering a physiologically relevant target for controlling neuronal excitability, modifying information flow, increasing physiological relevance and improving experimental reproducibility [56,82]. In summary, the expression of endogenous cholinergic tone and age-dependent developmental changes in cholinergic responsiveness in cortical cultures support the use of such cultures as physiologically useful models for the study of neural-robotic interfaces and other more conventional applications, and should not only be considered but actively exploited and further explored.

## Abbreviations

ACh: Acetylcholine; AChE: Acetylcholinesterase; ChAT: Choline acetyltransferase; CNS: Central nervous system; DIV: Days *in vitro*; DNA: Deoxyribonucleic acid; dNTPS: Deoxynucleoside triphosphates; EMEM: Eagle’s minimum essential medium; i: immature; LTP: Long term potentiation; m: Mature; mAChR: Muscarinic acetycholine receptor; mGluR: Metabotropic glutamate receptor; MCPG: (S)-α-methyl-4-carboxyphenylglycine; MEA: Multi-electrode array; MEC: Mecamylamine (nicotine hydrogen tartrate salt); nAChR: Nicotinic acetylcholine receptor; OXO-M: Oxotremorine methiodide; PBS: Phosphate buffered saline; RNA: Ribonucleic acid; RT-qPCR: Reverse transcription quantitative polymerase chain reaction; sADP: Slow after-depolarizing potential; TrkA: Tyrosine kinase A.See also Table 1 for the list of abbreviations used to denote electrophysiological measures employed and their definitions.

## Competing interests

The authors declare they have no competing interests.

## Authors’ contributions

All experiments were performed in the laboratory of Dr B. J. Whalley, University of Reading, UK. Experimental conception, design and analysis were performed by Dr M. Hammond and Dr B. J. Whalley. Dr M. Hammond, Dr B. J. Whalley and Dr A. Constanti prepared the manuscript. Immunocytochemical staining and imaging were conducted by Dr M. Hammond and Dr G. Bucci. The manuscript was appraised, revised and approved by Prof. K. Warwick, Dr S. J. Nasuto, Dr V. Becerra, Dr G. Bucci, Dr D. Xydas and Dr J. Downes. All authors read and approved the final manuscript.
